# Extreme sacrifice: sudden cardiac death in the US Fire Service

**DOI:** 10.1186/2046-7648-2-6

**Published:** 2013-02-01

**Authors:** Denise L Smith, David A Barr, Stefanos N Kales

**Affiliations:** 1 Health and Exercise Sciences, Skidmore College, 815 North Broadway, Saratoga Springs, NY 12866, USA; 2 University of Illinois Fire Service Institute, 11 Gerty Drive, Champaign, IL 61820, USA; 3 Harvard School of Public Health, Harvard Medical School, Occupational Medicine, Cambridge Hospital, Macht Building Suite 427, 1493 Cambridge Street, Cambridge, MA, 02139, USA

**Keywords:** Firefighting, Cardiovascular disease, Sudden cardiac death

## Abstract

Firefighting is a hazardous profession which has claimed on average the lives of 105 US firefighters per year for the past decade. The leading cause of line-of-duty mortality is sudden cardiac death, which accounts for approximately 45% of all firefighter duty-related fatalities. Strenuous physical activity, emotional stress, and environmental pollutants all strain the cardiovascular system, and each can increase the risk of sudden cardiac events in susceptible individuals. Sudden cardiac death is more likely to occur during or shortly after emergency duties such as fire suppression, despite the fact that these duties comprise a relatively small proportion of firefighters' annual duties. Additionally, cardiac events are more likely to occur in firefighters who possess an excess of traditional risk factors for cardiovascular disease along with underlying atherosclerosis and/or structural heart disease. In this review, we propose a theoretical model for the interaction between underlying cardiovascular disease in firefighters and the multifactorial physiological strain of firefighting.

## Review

### Introduction

It is widely recognized that firefighting is a hazardous profession. Many people outside the fire service mistakenly assume that most on-duty deaths result from burns and/or smoke inhalation. However, in the USA, the leading cause of duty-related deaths in firefighters is sudden cardiac death (SCD). In fact, cardiovascular disease (CVD) accounts for approximately 45% of all firefighter duty-related fatalities [1]. Currently, there are over 1 million firefighters in the USA, of whom approximately 70% are volunteers and 30% are career firefighters [2]. In proportion to their numbers, line-of-duty fatalities are similar with respect to frequency and etiology in volunteer firefighters and their career counterparts [3]. Sudden cardiac death and other CVD events are most likely to occur during the strenuous emergency duty of fire suppression, despite the fact that this duty comprises only 1%–5% of total annual working time spent during all fire service duties [4].

For every duty-related SCD event, almost 17 non-fatal cardiovascular events (heart attacks and strokes) occur in other firefighters during the course of their duties [5]. Given the team dynamics associated with firefighting, on-duty adverse health events can jeopardize job performance and safety of co-workers as well as the affected individual and may compromise public safety. Furthermore, firefighter fatalities also impose a significant economic burden on fire departments and local communities. Therefore, understanding the interaction of potential firefighting-related triggers of SCD and other CVD events with underlying heart disease conditions prevalent among firefighters is important for reducing the incidence of CVD events and is of major importance to fire departments, the medical community, and society as a whole.

In this review, we will summarize the factors producing marked cardiovascular strain on firefighters during firefighting activity. We will explore leading hypotheses and provide a theoretical model to explain how the strain of firefighting interacts with underlying CVD to precipitate a CVD event in vulnerable individuals in the US Fire Service.

### Acute cardiovascular strain of fire suppression activities

Firefighting leads to a rapid increase in heart rate initiated by sympathetic physiologic arousal from the fire alarm bell. Subsequently, maximal or near-maximal heart rates are achieved during physically strenuous firefighting activities. Perhaps of more functional significance, our work has shown a reduction in stroke volume following firefighting activity. We have reported a 35% reduction in stroke volume (using Doppler echocardiography) following short-term firefighting activity [6]. We have also documented a reduction in plasma volume (−14.8%) following 18 min of firefighting. Hypovolemia detrimentally affects heart function and increases blood viscosity. Specifically, we have found an increase in platelet number and function and alterations in partial thromboplastin time and fibrinogen levels following short-term firefighting activities [7,8]. These changes suggest a pro-coagulatory state which may increase the risk of thrombus formation.

We have recently reported that firefighting activity leads to an increase in arterial stiffness [9]. Furthermore, obese firefighters have an increased arterial stiffness at rest compared to non-obese firefighters [10]. In a study that involved 3–4 training drills, each lasting approximately 20 min and occurring over a 2.5- to 3.0-h time period, we found evidence of diastolic dysfunction (19% reduction in lateral wall E wave) post firefighting training [11].

**Figure 1:**
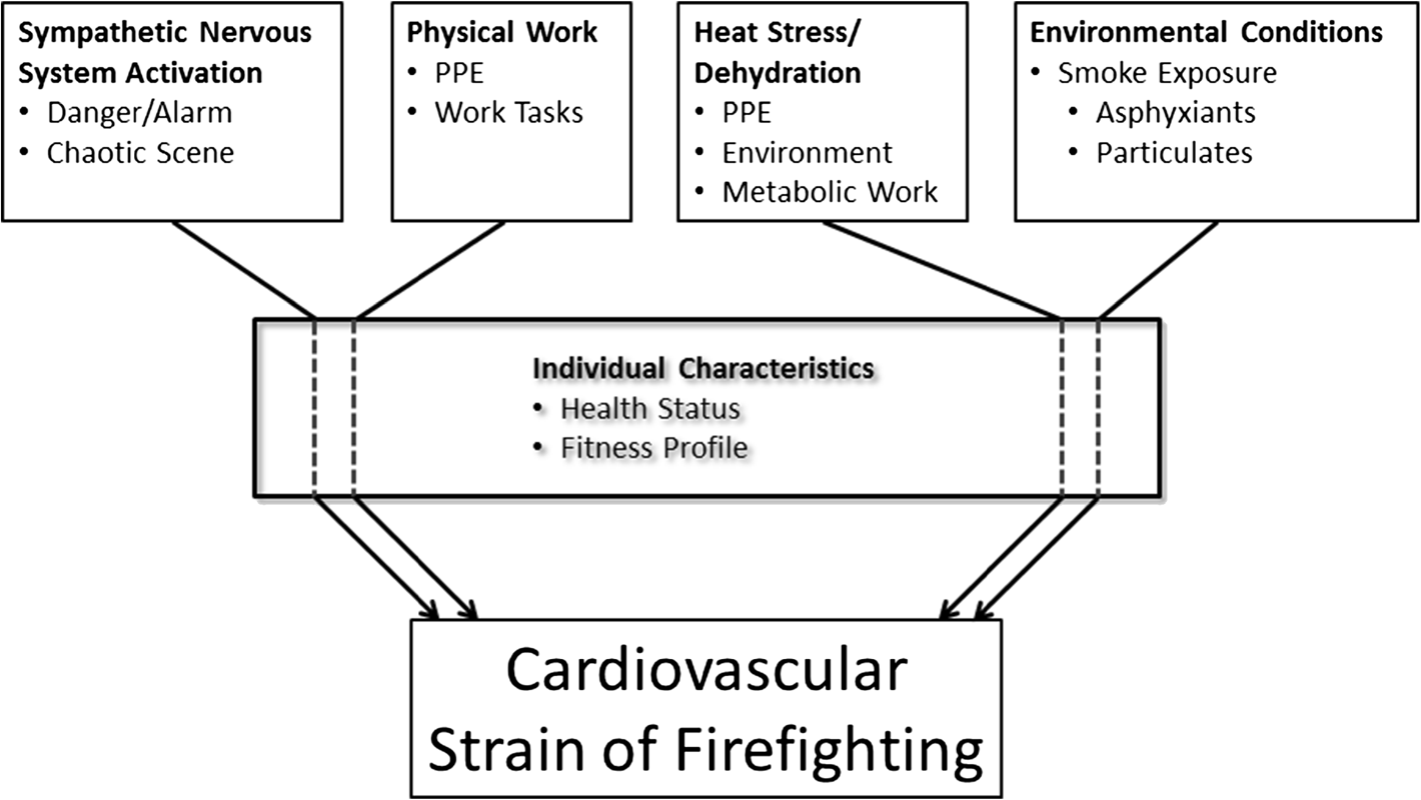
**Factors affecting the cardiovascular strain associated with firefighting. **PPE, personal protective equipment.

As shown in Figure 1, the magnitude of the cardiovascular strain of firefighting is the result of several interacting factors, including the degree of sympathetic nervous system activation, the physical work performed, the severity of heat stress and dehydration that the firefighter experiences, and the exposure to environmental conditions and pollutants contained in fire smoke. Furthermore, the magnitude of the cardiovascular strain is mediated by individual characteristics [12] such as health status [13] and physical fitness [14].

#### Sympathetic nervous system activation

During an emergency fire call, cardiovascular strain begins with initial activation of the sympathetic nervous system following the sound of an alarm bell [15]. Several research groups have reported marked increases in heart rate responses immediately following an initial alarm [15-17]. The importance of the sympathetic response in contributing to sudden cardiac events is highlighted by the finding that alarm response has been demonstrated in three independent studies to carry a five- to sevenfold risk of sudden cardiac death versus non-emergencies [4,18,19]. During this initial period, elevations in heart rate are likely due to significant activation of the sympathetic nervous system as there is little physical work and no significant exposure to high ambient temperatures.

Following an initial fire alarm, the sympathetic response continues during transport to the scene and at the fire scene. The fire scene is a chaotic environment characterized by loud noise, multiple auditory signals [20], and competing sensory inputs. Smoke often creates a dark operating environment, and the face mask and helmet can further decrease visual inputs, which may result in relative disorientation. The sensory stimuli and many life-threatening hazards promote the ‘fight or flight’ response, causing sympathetic activity to remain elevated or increase. Thus, sympathetic nervous system activation is a crucial mediator of altered physiology during firefighting activities.

#### Physical work

During structural firefighting, firefighters are regularly required to perform activities combining static and aerobic exertion such as stair and ladder climbing (while carrying heavy equipment), forcible entry, victim search and rescue, building ventilation, and fire attack and suppression. Moreover, these essential tasks are performed while wearing heavy (≥25 kg), multiple-layered and fully encapsulating protective clothing, which add to the metabolic demands of firefighting. For example, von Heimburg and co-workers [21] reported that transporting equipment up a six-story building in firefighting personal protective equipment (PPE) (including self-contained breathing apparatus (SCBA)) required 88% of a firefighter's maximum oxygen uptake. Although it is widely acknowledged that different firefighting tasks require different levels of energy expenditure, several studies and expert consensus have concluded that firefighting is a strenuous work requiring a minimum of 42 ml·kg^−1^·min^−1^ (or 12 metabolic equivalents (METs)) to perform the essential tasks safely [21,22].

#### Heat stress/dehydration

The most obvious physiological changes in response to performing heavy work while wearing encapsulating PPE in a hot environment are elevation of body temperature coupled with muscular and cardiovascular fatigue. We have documented an increase in core temperature of approximately 1.5°C (peaking at 38.6°C) following short-term (18 min) firefighting activity in a structure that contained live fires [23] and an increase of approximately 0.7°C (peak, 38.2°C) following 15 min of treadmill walking while wearing firefighting gear [24]. Similarly, Carter and co-workers [25] reported that 20 min of firefighting activity led to an increase in rectal temperature (+1.5°C). Importantly, core temperature continued to rise even after firefighters had ceased working and were removed from a hot environment (achieving a peak value of 38.9°C). Thus, heat stress is a major risk factor in fires and one that exacerbates the cardiovascular strain experienced by firefighters.

Strenuous work in a hot environment while wearing protective clothing also leads to profuse sweating and resultant dehydration. During strenuous firefighting simulations, firefighters have been reported to experience sweat rates up to and greater than 2 l∙ h^−1^[26,27]. Even when fluid ingestion is encouraged during rest periods between prolonged firefighting activities that include four–five evolutions, firefighters have been shown to experience levels of dehydration equivalent to 1.1% of body mass loss [11].

#### Environmental conditions

Despite the use of self-contained breathing apparatus, firefighters are routinely exposed to fire smoke, which contains toxic gases such as carbon monoxide (CO) and cyanide. When inhaled, CO diffuses through the alveolar–capillary membrane and binds with hemoglobin, forming carboxyhemoglobin. The formation of carboxyhemoglobin reduces the availability of hemoglobin to transport oxygen to the tissues, also binds cardiac and skeletal muscle myoglobin, and alters the intercellular use of oxygen, which can all result in tissue hypoxia [28]. Tissue hypoxemia due to CO and other asphyxiants (e.g., cyanide and hydrogen sulfide) may lead to myocardial ischemia in susceptible individuals [29].

Fire smoke also contains particulate matter associated with the promotion of arrhythmias, decreased heart rate variability, and increased blood pressure [30-32]. Other potential mechanisms to explain increased morbidity and mortality due to coronary heart disease (CHD) following modest exposures to smoke particulates include increased formation of free radicals leading to activation of proinflammatory and prothrombotic pathways [33]. In turn, this activation may result in endothelial dysfunction, increased blood coagulability, and accelerated progression of atherosclerosis.

#### Individual characteristics

The factors discussed above all interact with individual characteristics such as health status and fitness level to determine the magnitude of the cardiovascular strain of firefighting and whether responses to the strain are limited to physiologic adaptations or result in the activation of pathophysiologic pathways. Underlying cardiovascular health status is largely determined by fitness and the prevalence of cardiovascular risk factors among firefighters. In recent studies, the prevalence of smoking among general cohorts of firefighters was 10%–18%; however, the proportion of smokers among firefighters who suffered a CHD fatality while on duty is 40%–50% [19,34]. The prevalence of obesity and overweight is a growing concern in the fire service, ranging from 32% to 40% for obesity and 77% to 90% for combined overweight and obesity [35-39]. Approximately 20%–30% of firefighters have hypertension, and these numbers are expected to increase due to the increasing rate of obesity [10,40]. Furthermore, among firefighters with hypertension, the condition is often not well controlled [41]. Finally, dyslipidemia has been reported in greater than 20% of firefighters [42,43].

Given the strenuous nature of firefighting and the widespread concern about sudden cardiac death in the fire service, a high aerobic fitness level is clearly desirable. Yet, it is widely reported [36,44,45] that many firefighters do not meet the aforementioned aerobic fitness requirement of 42 ml·kg^−1^·min^−1^, indicating that, in many cases, a mismatch occurs between the fitness requirements of the job and the fitness profile of the firefighter. This is despite the fact that firefighters perceive themselves to have high fitness levels [46]. Lower levels of cardiorespiratory fitness are associated with metabolic syndrome and unfavorable metabolic profiles [42,47,48] as well as markedly increased risk of pathologic changes (ST segment change, dysrhythmia, and abnormal heart rate recovery) during peak exercise [49]. Conversely, high fitness levels decrease these risks. Such findings demonstrate the importance of a high aerobic capacity not only for the energy demands of this occupation but also because of a likely protective effect against SCD and other CVD events in this population.

### Epidemiology of CVD events in firefighters

A typical firefighting shift is characterized by long periods of low-intensity work interspersed with occasional bouts of moderate to high-intensity activity [50]. As many firefighters spend a lot of time in sedentary activities, it is not surprising that in large part they do not differ from the overall working population in terms of aerobic fitness, body composition (obesity), and other cardiovascular risk factors. It has been shown that duty-related CVD events occur almost exclusively in firefighters who possess a clustering of traditional risk factors for CVD and some type of underlying structural heart disease [18,19,34,40]. The vast majority of SCD in firefighters (90%) is attributable to CHD. Most specifically, autopsies on firefighters who have suffered SCD most often show coronary atherosclerosis usually accompanied by left ventricular hypertrophy (LVH)/cardiomegaly [18,19,34]. Studies show that about 25%–30% of CHD-related firefighting fatalities occur in persons who have a previously known diagnosis of CHD or a clinical equivalent (e.g., peripheral artery disease, ischemic stroke, etc.). In firefighters who suffer SCD without significant CHD, autopsies are more likely to reveal cardiomegaly due to hypertensive heart disease or idiopathic cardiomyopathy and, less frequently, significant valvular anomalies.

Clinical CVD, subclinical CVD, and the presence of specific CVD risk factors increase the risk of sudden cardiac events. Studies investigating the prevalence of cardiovascular risk factors in firefighters suggest that firefighters often have risk factor prevalence similar to the general population [18,19,40,51]. Table 1 identifies the relative risk of cardiovascular outcome by risk factor based on studies that investigated on-duty CHD fatalities, non-CHD cardiovascular-related retirements, and CHD retirements [18,19].

**Table 1:** Relative risk of cardiovascular outcome by risk factor in firefighters

	**On-duty CHD fatalities**	**Non-CHD cardiovascular retirements**	**CHD retirements**
	**OR (95% CI)**[19]	**OR (95% CI)**[18]	**OR (95% CI)**[18]
Current smoking	8.6 (4.2–17)	2.5 (1.2–5.1)	3.9 (2.5–6.2)
Hypertension	12 (5.8–25)	11 (6.1–20)	5.4 (3.7–7.9)
Obesity, BMI ≥ 30 kg·m^−2^	3.1 (1.5–6.6)	3.6 (2.0–6.4)	1.4 (0.96–1.93)
Cholesterol ≥ 5.18 mmol·l^−1 ^(200 mg·dl^−1^)	4.4 (1.5–13)	1.1 (0.51–2.24)	2.4 (1.6–3.6)
Diabetes mellitus	10.2 (3.7–28)	7.7 (2.9–20)	13 (6.1–28)
Prior diagnosis of CHD	35 (9.5–128)	NA	30 (9.1–96)
Age ≥ 45 years old	18 (8.5–40)	26 (13–51)	63 (35–111)

Hypertension is defined as resting blood pressure ≥ 140/90 mmHg, previous diagnosis of hypertension, or receiving antihypertensive therapy. Diabetes mellitus is defined as random blood glucose > 8.3 mmol·l^−1 ^(150 mg·dl^−1^), previous diagnosis, or receiving insulin or hypoglycemic medication. CHD, coronary heart disease; OR, odds ratio; CI, confidence interval; BMI, body mass index.

#### Factors associated with sudden cardiac events

Numerous studies have shown that a variety of stressful situations can trigger acute CVD events, particularly in susceptible individuals with underlying CHD. Heavy physical exertion [52-54] or strenuous work [55] can trigger the onset of acute CVD events, particularly in sedentary individuals. Emotional stress, such as episodes of excitement and frustration/anger, has also been shown to be associated with triggering CVD events in individuals with known CHD [56]. Environmental insults such as surges in air pollution [32] or influenza activity [57] can also precipitate sudden CVD events in susceptible individuals.

#### Firefighting as a trigger for SCD and other CVD events

The three factors highlighted above (physical exertion, emotional stress, and environmental pollutants) are encountered by firefighters on a regular basis during fire suppression activities. Therefore, it is likely that these multiple stressors may function independently or work in concert to precipitate CVD in firefighters depending on the conditions encountered and the individual firefighter's susceptibility. As shown in Table 2, three independent studies have provided compelling evidence that strenuous firefighting activities can precipitate cardiovascular events in susceptible firefighters [4,18,19]. Together, these studies show remarkably similar odds for SCD and other acute CVD events during emergency firefighting activities compared to non-emergency duties. The largest of these three studies [4] investigated each of the 449 line-of-duty deaths from CHD that occurred between 1994 and 2004. From the three different risk estimates—calculated to account for uncertainties in the relative time spent by firefighters across different duties—the risk of CHD-related deaths remained markedly elevated for the same strenuous firefighting activities. For example, although fire suppression duties (mitigating and extinguishing fires) were found to represent between 1% and 5% of a firefighter's annual working time, fire suppression activity accounted for more than 30% of line-of-duty CHD deaths. Statistically, this translated to a relative risk of SCD during fire suppression of roughly 10 to 100 times the risk encountered during non-emergency duties [4]. Notably, strenuous fire suppression activities are associated with a much higher risk of CHD death than exercise alone, which may be explained by the added cardiovascular strain of emotional and environmental stressors.

**Table 2:** Duty-specific risks of cardiac events in firefighters

**Type of duty**	**Relative risk of CHD death**[19]	**Relative risk of cardiac event resulting in early retirement**[18]	**Relative risk of CHD death**[4]
Fire suppression (OR (95% CI))	64.1 (74–556)	51 (12–223)	53 (40–72)
Physical training (OR (95% CI))	7.6 (1.8–31.3)	0.68 (0.2–2.7)	5.2 (3.6–7.5)
Alarm response (OR (95% CI))	5.6 (1.1–28.8)	6.4 (2.5–17)	7.4 (5.1–11)
Alarm return (OR (95% CI))	3.4 (0.8–14.7)	0.37 (0.07–1.8)	5.8 (4.1–8.1)
EMS and other emergencies (OR (95% CI))	1.7 (0.5–5.9)	0.75 (0.3–1.8)	1.3 (0.9–2.0)
Firehouse and other non-fire emergencies	1.0	1.0	1.0

CHD, coronary heart disease; OR, odds ratio; EMS, emergency medical service; CI, confidence interval.

#### Theoretical model for sudden cardiac events in the fire service

**Figure 2:**
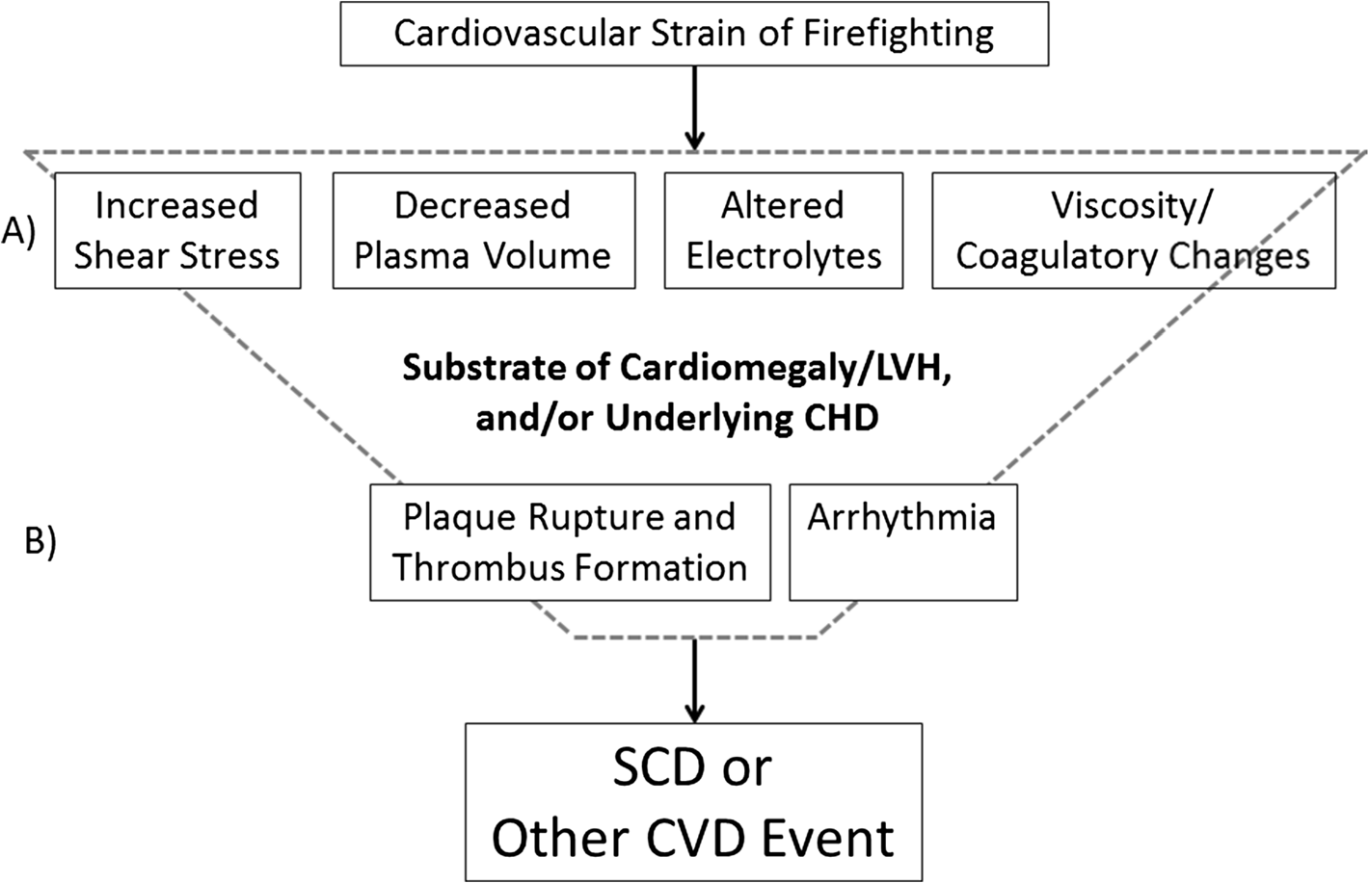
**Theoretical model of sudden cardiac events in firefighters. **LVH, left ventricular hypertrophy; CHD, coronary heart disease; SCD, sudden cardiac death; CVD, cardiovascular disease.

Sudden cardiac death in firefighters most likely arises from a multifaceted interplay of substrates and triggers. As seen in Figure 1, the combination of sympathetic nervous system activation, strenuous physical work, heat stress/dehydration, and environmental conditions leads to significant cardiovascular strain which is mediated by multiple individual characteristics. Figure 2 (row A) depicts several interrelated cardiovascular changes that are known to occur as a result of firefighting activity, including increases in heart rate and blood pressure leading to increased shear stress, a decrease in plasma volume and resulting change in electrolyte concentration, and an increase in viscosity and coagulatory changes. These alterations in physiological function may range from moderate to severe depending upon the specifics of the specific emergency scene encountered by firefighters but do not normally pose a great risk in healthy individuals. However, in individuals with underlying structural CVD, the physiological responses to firefighting may lead to one or more pathological changes that greatly increase the risk of plaque rupture and thrombus formation and/or arrhythmia (row B), which can lead to SCD or another acute CVD event.

### Causes of sudden cardiac death

Although a series of complex pathophysiologic processes are involved in SCD, pump failure usually due to cardiac arrhythmias is ultimately the cause of death. Sudden cardiac death can be caused by many conditions; however, a very large percentage of SCD is caused by CHD (i.e., atherosclerosis) and/or cardiomegaly/LVH. CHD is associated with stenosis, ischemia, plaque rupture, and the formation of an occlusive thrombus. Cardiomegaly/LVH is associated with increased risk of fatal arrhythmias.

#### Disorders of the coronary arteries

Coronary heart disease and its consequences account for approximately 80% of all SCD in Western countries [58-61]. However, anomalous coronaries, coronary embolism, and coronary arteritis are all important recognized causes of SCD. Patients with anomalous coronaries are more prone to sudden cardiac death with physical exertion and dehydration, which may play a small but significant role in SCD in young firefighters.

Atherosclerosis has long been associated with the gradual and cumulative accumulation of lipid in the arterial wall. Both intravascular ultrasound and wartime autopsy studies provide strong evidence of a high prevalence of subclinical coronary atherosclerosis in young and middle-aged Americans [62]. More recent understanding of atherosclerotic disease emphasizes the role of inflammatory mediators in the initiation and progression of the disease. Atherosclerotic plaque accumulates in the vessel wall in a non-linear fashion over a period of many decades [63]. Plaque rupture exposes the platelets and coagulatory factors to prothrombotic elements in the vessel way, which initiates a thrombotic response. The newly formed thrombus may cause sudden flow obstruction, leading to an acute coronary syndrome. A non-occlusive or transiently occlusive thrombus most frequently underlies acute coronary syndromes (ACS) without ST segment elevation (non-ST segment elevation myocardial infarction (NSTEMI)). In contrast, a stable and occlusive thrombus is associated with STEMI.

Many plaque-rupturing events, which cause ACS, occur on thin vulnerable plaques and not necessarily on hemodynamically significant stenoses. Hemodynamically significant stenoses *per se* are not a pre-requisite for ACS but are an indication of a higher overall plaque burden and, thus, a greater likelihood of having an ACS event. The mechanisms linking a previously quiescent atherothrombotic plaque to an acute coronary syndrome are complex and include factors related to plaque stability and hemostatic balance. Furthermore, there is evidence that external triggers, such as heavy work and emotional stress, may lead to acute changes that precipitate a cardiac event [64]. Studies that have examined autopsy findings of firefighters who suffered SCD have found that the majority of victims had evidence of coronary atherosclerosis [19,34].

#### Cardiomegaly and left ventricular hypertrophy

Cardiomegaly (increased heart size and mass) and LVH (increased wall thickness and mass) are structural abnormalities that are associated with SCD. Left ventricular hypertrophy has been shown to be a powerful predictor of cardiovascular morbidity and mortality in population-based studies [65-68]. The risk of sudden death is approximately six times greater for men with electrocardiogram-detected LVH than men without LVH [69]. Furthermore, a strong-graded association between left ventricular mass and increased cardiovascular risk has been demonstrated [70]. Several studies have confirmed that the increased risk associated with LVH is independent of other factors such as age, gender, smoking status, diabetes, and serum cholesterol [71-73] and have shown the progressive association between increased left ventricular mass and cardiovascular morbidity and mortality [71,72]. In a majority of cases, LVH is typically a result of hypertension and/or coronary disease. In the absence of hypertension or coronary disease, the main cause of LVH is cardiomyopathy, which often carries a higher risk of SCD. Although the precise mechanisms by which LVH causes CV morbidity and mortality are not fully understood, more recent work has suggested that myocardial fibrosis plays an important role. Left ventricular hypertrophy is frequently associated with fatal arrhythmias and likely contributes to the pathophysiology of SCD when present [58]. However, the precise mechanisms by which LVH promotes cardiovascular morbidity and mortality are not completely understood.

Hypertrophic cardiomyopathies have long been associated with SCD in athletes, and recently, Rowland has raised the provocative question about the arrhythmogenic nature of LVH that accompanies athletic training [74]. Although it has long been recognized that individuals dying of CHD tend to have heavier hearts than those dying of non-cardiac causes [61], the prevalence and relevance of cardiomegaly/LVH have received far less attention than has the presence of atherosclerosis and stenosis. Significantly, a recent study that retrospectively studied the cardiac findings of adults who died of SCD attributed the deaths to cardiomegaly/LVH, CHD, or both [75]. This study found that cardiomegaly/LVH is a frequent cause of SCD in the general public and is highly associated with obesity and death at a younger age than CHD. A review of on-duty firefighting CHD fatalities has also documented that close to 60% of victims had evidence of LVH/cardiomegaly at autopsy [34].

There is mounting evidence that LVH/cardiomegaly is common among US firefighters and plays a major role in CVD events in the fire service. In a seminal 2003 case–control investigation of on-duty CHD fatalities, we found evidence for LVH in 76% of the CHD deaths where the autopsy results were available [19]. In a larger follow-up case–fatality study, we compared firefighters succumbing to on-duty CHD fatalities with firefighters suffering non-fatal CHD events leading to retirement. Among the fatalities, LVH/cardiomegaly was mentioned in summary reports of almost 60% of the available autopsies [34]. Both of these studies suggested chronically uncontrolled hypertension, the major risk factor for LVH, among the majority of the fatal CHD cases.

This review has proposed a theoretical model for the interaction between underlying cardiovascular disease in firefighters and the multifactorial strain of firefighting. We have posited that both atherosclerotic heart disease and left ventricular hypertrophy/cardiomegaly are important substrates that markedly increase the risk of sudden cardiac death. Additional research is necessary to understand the risk associated with a given level of atherosclerotic burden, cardiomegaly, or left ventricular hypertrophy. Furthermore, research is necessary to identify when and how firefighters should be screened for these conditions.

## Conclusions

Firefighting activities involve sympathetic arousal, heavy strenuous work, and adverse environmental conditions that can lead to hyperthermia and dehydration with considerable associated cardiovascular strain on firefighters. The theoretical model proposed in this article is based on diverse lines of evidence, including physiological studies of firefighters during strenuous emergencies, epidemiologic studies linking CVD event risk to specific types of duty, as well as autopsy data confirming the presence of underlying heart disease in almost all victims. The model suggests that in susceptible individuals with underlying structural heart disease (most often CHD and LVH), the cardiovascular strain associated with firefighting may trigger a sudden cardiac event through several biological pathways. Increases in shear stress may cause rupture of vulnerable plaque, resulting in occlusion of coronary arteries, and this may be exacerbated by hyper-coagulability which increases the risk of thrombotic events. Ischemia (due to an increase in myocardial oxygen demand) may exceed myocardial demand, resulting in electrical, mechanical, and biochemical dysfunction of the cardiac muscle, precipitating fatal arrhythmias. Changes in electrolytes and exposure to environmental conditions (such as gaseous and particulate toxicants in smoke) may also increase susceptibility to arrhythmias [30-32], particularly in those with LVH and other forms of cardiomegaly.

## Abbreviations

ACS: acute coronary syndromes; CHD: coronary heart disease; CO: carbon monoxide; CVD: cardiovascular disease; LVH: left ventricular hypertrophy; METs: metabolic equivalents; NSTEMI: non-ST elevation myocardial infarction; PPE: personal protective equipment; SCBA: self-contained breathing apparatus; SCD: sudden cardiac death; STEMI: ST elevation myocardial infarction.

## Competing interests

SNK has served as an expert witness in legal cases involving firefighters and was also contracted to revise the Heart Disease Manual of the International Association of Fire Fighters. DLS has served as an expert witness in legal cases involving firefighters. The other author has no competing interests to declare.

## Authors’ contributions

DLS conceived of the review and collaborated with SNK on the development of the theoretical framework for the manuscript. DLS provided an initial draft of the manuscript. DAB provided significant review and revision to the manuscript draft. SNK reviewed and provided significant contributions to the final manuscript. All authors read and approved the final manuscript.
